# Supplementary material for: Getting there in one piece: The Rac pathway prevents cell fragmentation in a nonprotrusively migrating leader cell during organogenesis
Source: bioRxiv. 2023 Dec 4:2023.12.01.569642. Preprint. [Version 2] doi: 10.1101/2023.12.01.569642 (PMC10723291; doi:10.1101/2023.12.01.569642)
Supplement: Supplement 1 [file NIHPP2023.12.01.569642V2-supplement-1.pdf]

bioRxiv preprint doi: https://doi.org/10.1101/2023.12.01.569642; this version posted December 4, 2023. The copyright holder for this preprint (which was not certified by peer review) is the author/funder, who has granted bioRxiv a license to display the preprint in perpetuity. It is made available under aCC-BY 4.0 International license.

Figure S1. DTC-specific knockdown of *mig-2* Rac1 GTPase but not parallel cell-engulfment pathway *ced* genes causes DTC cell morphology and migration/bifurcation defects.

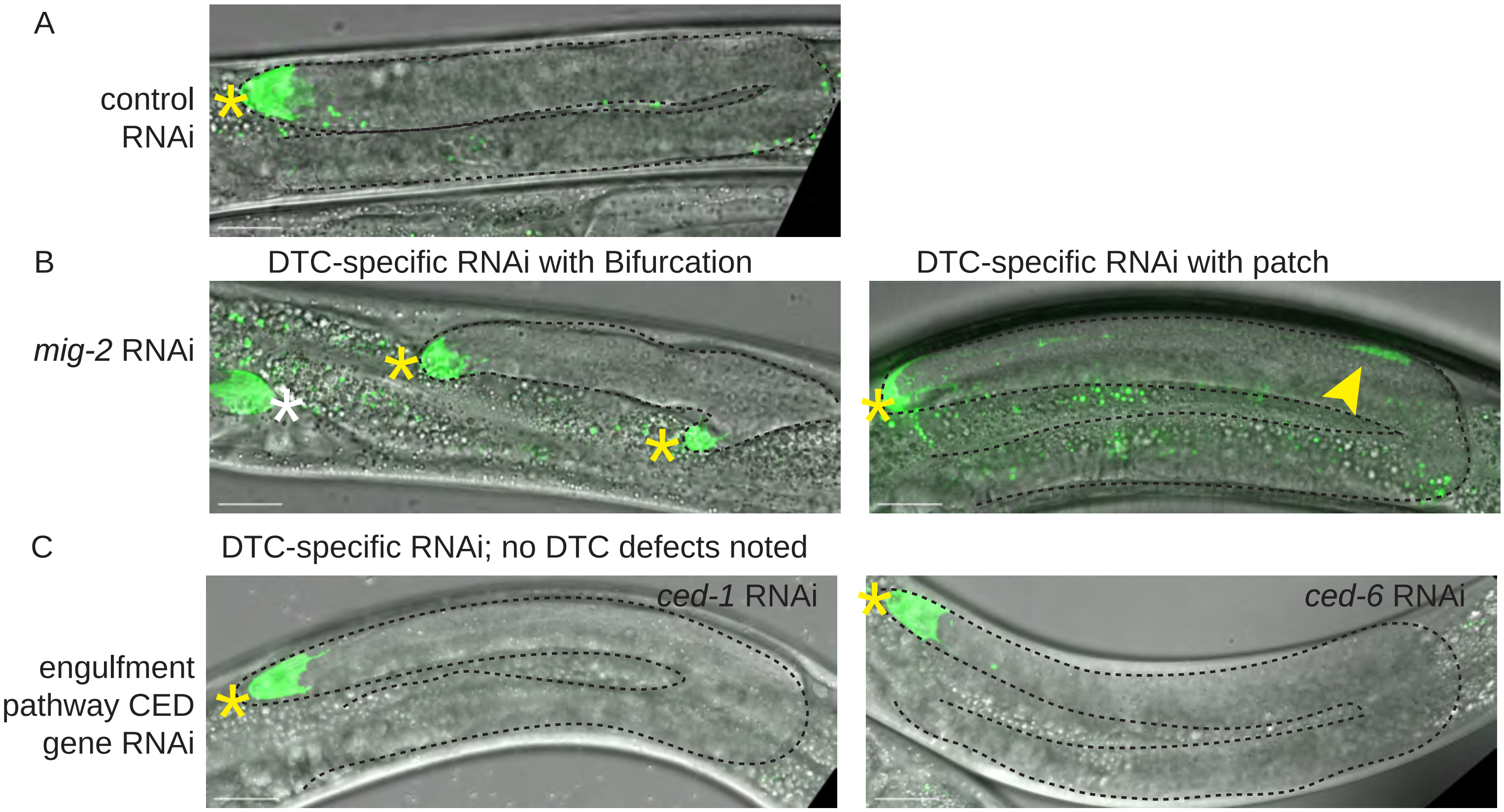

Figure S1. DTC-specific knockdown of *mig-2*/RhoG GTPase, but not parallel cell-engulfment pathway *ced* genes causes DTC cell morphology and migration/bifurcation defects. Strain expressing a combined *lag-2* promoter-driven membrane marker and *rde-1* rescue transgene *lag-2p::mNG::PLC*$^{\delta PH}$*::F2A::rde-1* in a genetic background that is *rde-1(ne219)* loss of function and *rrf-3(pk1426)* hypersensitive to RNAi. **(A)** On control RNAi (empty vector L4440), one site of strong mNG expression is visible in each gonad arm in late L4 hermaphrodites–the distal tip cell. No expression is observed near the bend of the gonad (inset). **(B)** DTC-specific RNAi knockdown of the *mig-2/*RhoG gene causes a range of cellular and anatomical gonad defects: gonad bifurcation (left), in which mNG expression is always observed on both tips, and the formation of a second "patch" of mNG expression near the bend of an otherwise anatomically normal gonad (right). N=34 total RNAi-treated animals scored, 7/34 had second patch only and an additional 2/34 had a bifurcated gonad. *mig-2* acts upstream of the Rac pathway *ced* genes[40]. **(C)** DTC-specific knockdown of *ced-1* (N=33) and *ced-6* (N=29), members of a parallel engulfment pathway to *ced-10, ced-5, ced-2,* and *ced-12.* No DTC or gonad morphology defects were observed, though in 3/62 samples a delay in dorsal elongation was noted in a single gonad arm. Maximum projection of GFP fluorescence channel through all Z-slices with mNG signal merged with maximum projection of DIC image through slices capturing the gonad tip(s). Imaged at late L4 stage. Visible gonad outlined in black dashed line. Yellow asterisks mark gonad tips of focal gonads, white asterisk marks tip of other gonad arm, yellow arrowhead marks the patch. Autofluorescence of the gut is visible as green punctae; this is unrelated to expression of the fluorescent protein. Scale bars 20 μm.

bioRxiv preprint doi: https://doi.org/10.1101/2023.12.01.569642; this version posted December 4, 2023. The copyright holder for this preprint (which was not certified by peer review) is the author/funder, who has granted bioRxiv a license to display the preprint in perpetuity. It is made available under aCC-BY 4.0 International license.

Figure S2. The nuclear signal is not always found in the distal-most tip.

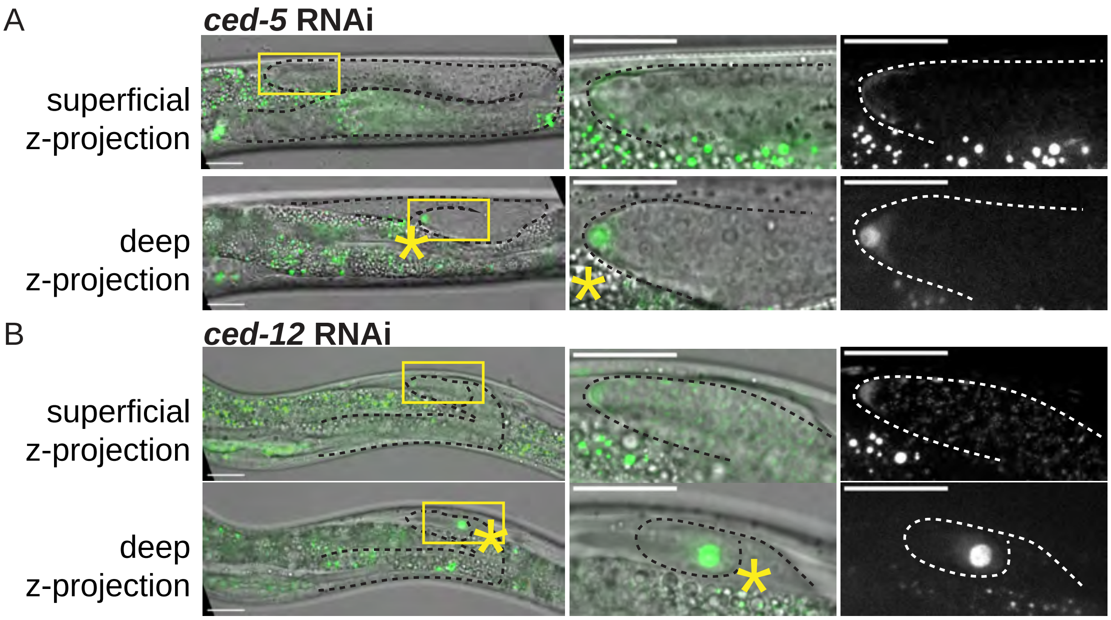

Figure S2. The nuclear DTC signal is not always found in the distal-most tip. (A) RNAi knockdown of *ced-5* in the strain bearing the *gfp*::*hlh-2* allele. Upper left, maximum projection of GFP fluorescence channel through the more superficial tip of a bifurcated gonad in GFP merged with DIC. Inset right, the more superficial tip has diffuse cytoplasmic GFP::HLH-2 signal, despite falling in the anatomically correct position of a gonad distal tip. Below, maximum projection of GFP fluorescence channel through the deeper tip of same bifurcated gonad merged with DIC. Inset right, the deeper tip has nuclear GFP::HLH-2 signal. N = 1/9 total bifurcated gonads imaged **(B)** RNAi knockdown of *ced-12* in the strain bearing the *gfp*::*hlh-2* allele. Upper left, maximum projection of GFP fluorescence channel through the more superficial tip of a bifurcated gonad in GFP merged with DIC. Inset right, the more superficial tip has diffuse cytoplasmic GFP::HLH-2 signal. Below, maximum projection of GFP fluorescence channel through the deeper tip of same bifurcated gonad merged with DIC. Inset right, the deeper tip has nuclear GFP::HLH-2 signal, despite having made an extra turn towards the viewer. N = 1/10 total bifurcated gonads imaged. Visible gonad outlined in black or white dashed line. Yellow asterisks mark gonad tip with DTC nucleus, yellow arrowhead marks the enucleate patch. Yellow boxes show position of insets in larger image. Autofluorescence of the gut is visible as punctae; this is unrelated to expression of the fluorescent proteins. Imaged at L4 stage. Scale bars 20 μm.

bioRxiv preprint doi: https://doi.org/10.1101/2023.12.01.569642; this version posted December 4, 2023. The copyright holder for this preprint (which was not certified by peer review) is the author/funder, who has granted bioRxiv a license to display the preprint in perpetuity. It is made available under aCC-BY 4.0 International license.

Figure S3. Bifurcated germlines begin differentiating at the second tip in L4 larva

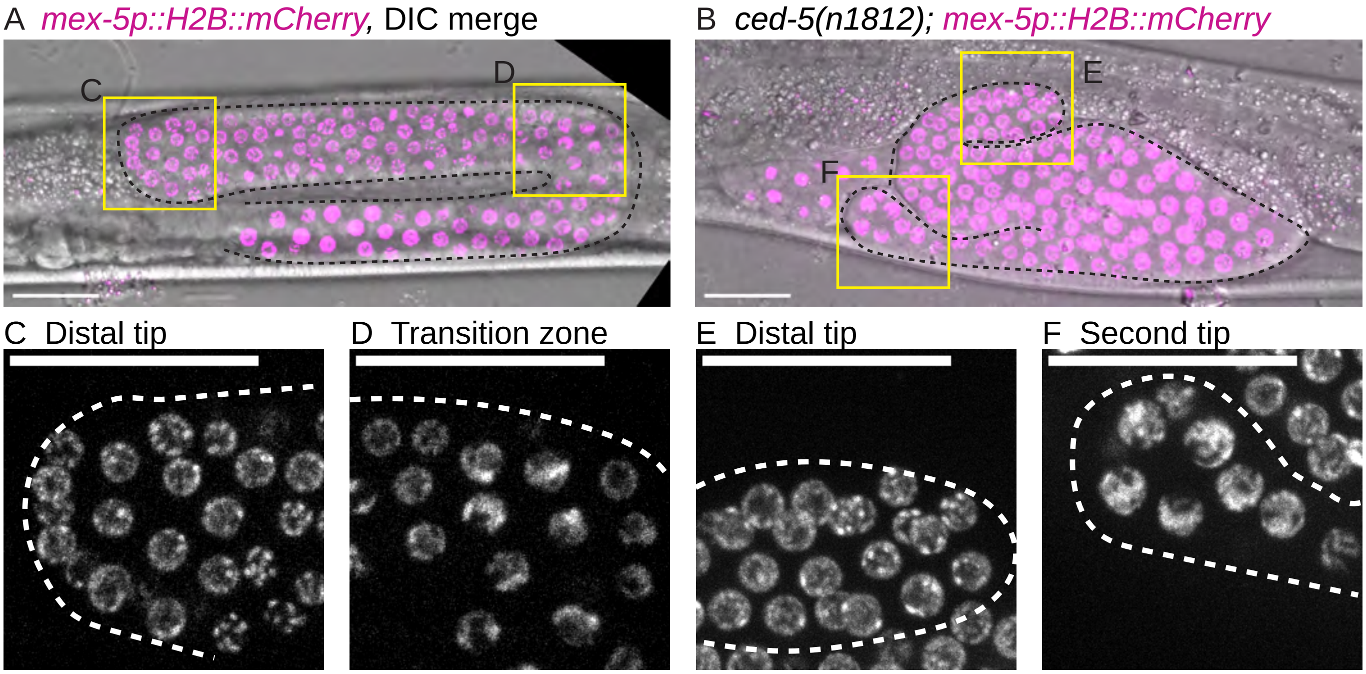

Figure S3. Bifurcated germlines are mispatterned and have differentiating germ cells at the distal end in the L4 stage. Micrographs comparing features of adult germlines between control (A, C, D) and *ced-5(n1812)* (B, E, F) animals. Germ cell nuclei visualized with the *naSi2(mex-5p::H2B::mCherry)* transgene[74], magenta in merge. **(A)** Z-projection through DIC image of L4 control gonad merged with germ cell histone fluorescence. Boxes show positions of insets that follow. **(B)** Z-projection through DIC image of L4 *ced-5(n1812)* gonad merged with germ cell histone fluorescence. Boxes show positions of insets that follow. **(C)** Enlargement of control distal tip fluorescence image showing undifferentiated germ cell nuclei. **(D)** Enlargement of the fluorescence image of the control meiotic transition zone showing its distinctive crescent-shaped nuclear morphology. **(E)** Enlargement of distal tip of *ced-5(n1812)* fluorescence image of gonad distal tip showing undifferentiated germ cell nuclei. **(F)** Enlargement of second tip of *ced-5(n1812)* mutant goand fluorescence image showing crescent-shaped germ cell nuclei characteristic of cells that have entered meiosis. Gonads outlined in black or white dashed lines. Autofluorescence of the gut is visible as punctae; this is unrelated to expression of the fluorescent proteins. Scale bars: 20 μm.
